# Multifaceted involvements of Paneth cells in various diseases within intestine and systemically

**DOI:** 10.3389/fimmu.2023.1115552

**Published:** 2023-03-13

**Authors:** Chenbin Cui, Xinru Wang, Lindeng Li, Hongkui Wei, Jian Peng

**Affiliations:** ^1^ Department of Animal Nutrition and Feed Science, College of Animal Science and Technology, Huazhong Agricultural University, Wuhan, China; ^2^ The Cooperative Innovation Center for Sustainable Pig Production, Wuhan, China

**Keywords:** Paneth cell, Crohn’s disease, necrotizing enterocolitis, liver disease, acute pancreatitis, COVID-19, therapeutic strategy

## Abstract

Serving as the guardians of small intestine, Paneth cells (PCs) play an important role in intestinal homeostasis maintenance. Although PCs uniquely exist in intestine under homeostasis, the dysfunction of PCs is involved in various diseases not only in intestine but also in extraintestinal organs, suggesting the systemic importance of PCs. The mechanisms under the participation of PCs in these diseases are multiple as well. The involvements of PCs are mostly characterized by limiting intestinal bacterial translocation in necrotizing enterocolitis, liver disease, acute pancreatitis and graft-vs-host disease. Risk genes in PCs render intestine susceptible to Crohn’s disease. In intestinal infection, different pathogens induce varied responses in PCs, and toll-like receptor ligands on bacterial surface trigger the degranulation of PCs. The increased level of bile acid dramatically impairs PCs in obesity. PCs can inhibit virus entry and promote intestinal regeneration to alleviate COVID-19. On the contrary, abundant IL-17A in PCs aggravates multi-organ injury in ischemia/reperfusion. The pro-angiogenic effect of PCs aggravates the severity of portal hypertension. Therapeutic strategies targeting PCs mainly include PC protection, PC-derived inflammatory cytokine elimination, and substituting AMP treatment. In this review, we discuss the influence and importance of Paneth cells in both intestinal and extraintestinal diseases as reported so far, as well as the potential therapeutic strategies targeting PCs.

## Introduction

Paneth cells (PCs) were first discovered by Gustav Schwalbe in 1872 (1) and named by Josef Paneth in 1887 (2). Acting as a unique type of intestinal epithelial cells, PCs are derived from adjacent intestinal stem cells (ISCs) and located at the base of epithelial crypt region. In small intestine, the differentiation of PCs is conducted under the condition of Notch signaling off and Wnt signaling on in ISCs (3). PCs can be identified by the presence of cytoplasmic abundant granules mainly including antibacterial peptides (AMPs), interleukin (IL)-17A, tumor necrosis factor (TNF)-α and CD95 ligand (4–6). There are many identified AMPs such as α-defensin, lysozyme, and regenerating islet-derived 3α (REG3α, REG3γ in mice) in PCs. PCs possess abundant endoplasmic reticulum (ER) and trans-Golgi network to realize their highly secretory nature (7). As a key component of intestinal innate immune, the functional PCs are of great importance for intestinal homeostasis. Abundant AMPs secreted by PCs control the balance of host-microbiota interactions within small intestine (8). The defects in PCs or AMP expression could lead to microbiota disorders and mucosal penetration by intestinal bacteria (9, 10). In addition, PCs support the functions of ISCs by providing several factors such as Wnt3a, epidermal growth factor (EGF), and metabolites (11–13). PCs can manipulate intestinal epithelial apoptosis by releasing CD95 ligand (14).

Considering that PCs maintain the health of intestine in multiple manners, PC dysfunction is generally involved in intestinal disorders and even diseases such as Crohn’s disease (CD) and necrotizing enterocolitis (NEC) (15). The impaired unfolded protein response (UPR) and autophagy turn PCs into an origin of intestinal inflammation (16). Furthermore, the mutations in several UPR- and autophagy-related genes in PCs such as autophagy related 16 like 1 (*ATG16L1*) and X-box-binding protein 1 (*XBP1*) are identified risk factors for CD (17). Additionally, NEC pathogenesis is associated with intestinal bacterial translocation (18).

Although the presence of PCs is mainly limited in small intestine under intestinal homeostasis, PCs participate in the pathogenesis of extraintestinal diseases in addition to intestinal diseases. Large amounts of extraintestinal diseases such as liver diseases, acute pancreatitis (AP) and graft-vs-host disease (GVHD) involve the decreases in PC number and AMP expression (19–21). PC defects result in visceral hypersensitivity that induced by the expansion of intestinal *Escherichia coli*, implying the susceptibility to diseases after PC disruption (22). Ischemia/reperfusion (IR)-induced multi-organ injury is mediated by the IL-17A secreted by PCs (6). Here we provide an overview of the influence and importance of PCs on various diseases within intestine and other bodily organs, as well as potential therapeutic strategies targeting PCs in these diseases.

## PCs in inflammatory bowel disease

Inflammatory bowel disease (IBD) is a severe intestinal disease in the 21st century all over the world (23). IBD is divided into two types, ulcerative colitis (UC) and CD (24). UC is a chronic and continuous disease impacting the colon (25), whereas CD is a transmural disease occurring anywhere in the gastrointestinal tract (from mouth to anus) (26). PC abnormalities are observed in 20%-50% of CD patients and are more prevalent in pediatric CD patients than adult CD patients (27, 28). The presence of PC abnormalities is used to forecast the recurrence of CD after surgery (29). Under healthy condition, mouse PCs are limited in ileum, while human PCs normally exist in ileum as well as sporadically in cecum and ascending colon (30). The decreased expressions of α-defensins (HD5 and HD6) are observed in ileal PCs of CD patients, which is attributed to the diminished Wnt ligands (enhancers of AMP expression) in monocytes and partly in ileum, highlighting the multiple regulations of PCs in CD (31).

Since PCs exhibit continuous AMP synthesis and release, functional mitochondria in PCs are required to provide energy. Recent studies have reported that active CD is associated with mitochondrial abnormalities in PCs, thus impairing PC function (32, 33). The importance of mitochondrial homeostasis in PCs is further confirmed by the fact that the level of Prohibitin 1 (PHB1), a major component protein of the inner mitochondrial membrane, is down-regulated in the mucosal biopsies from CD patients (34), and *Phb1* deficiency in PCs triggers PC defects and spontaneous ileitis in mice (35).

In addition to small intestine, PCs are occasionally observed at other sites under pathological condition, such as stomach and colon, and the phenomenon is called PC metaplasia (36). Both UC and CD patients display the occurrence of abundant metaplastic PC along the whole colon (30). It is generally accepted that metaplastic PCs tend to protect the intestine from infections. Metaplastic PCs secrete several AMPs into colonic lumen, such as α-defensins, lysozyme, sPLA2 and intelectin-2 (ITLN2), which is considered as a host defense response to IBD (37–40). The activities of AMPs in colon are associated with the degree of intestinal injury in IBD. Notably, colonic expression of HD5 is significantly higher in CD than in UC, indicating that HD5 may be a potential biomarker in IBD diagnosis, a complicated process with 30% misdiagnosis (40). However, lysozyme derived from metaplastic PCs has been proved to be detrimental to colon. Lysozyme-processed and non-processed *Ruminococcus gnavus* (a CD-associated pathobiont) induced distinct immune responses in colon (41). Pro-inflammatory responses are triggered by *Ruminococcus gnavus* after lysozyme processing, whereas the transfer of *Ruminococcus gnavus* to *Lyz1* knockout mice contributes to a type 2 immune response promoting intestinal epithelial repair (41). Considering that both CD and UC increase the risk of colorectal cancer (42), experimental studies on the role of colonic metaplastic PCs in this event should be conducted since PCs can secrete Wnt3a and EGF that might promote cancerization in intestine.

There are many identified risk genes of CD in PCs such as *ATG16L1*, *NOD2*, and *XBP1* (16, 17). Most of these risk genes are associated with the normality of autophagy and UPR, and ATG16L1 has been the most well-studied target so far. CD patients homozygous for the *ATG16L1* risk allele exhibit granule disruption and mitochondria degeneration in PCs (43). Besides, *ATG16L1* mutations also lead to ER stress in PCs as demonstrated by the enhanced levels of GPR78 and pEIF2α (44). The mechanism under elevated ER stress caused by *ATG16L1* deficiency is the impaired removal of IREα, an ER stress sensor (45). The protective role of ATG16L1 is further validated by the increased susceptibility to bacteria-induced inflammation in *ATG16L1*-mutated mice (46). *S. typhimurium*-induced ER stress triggers ATG16L1-mediated secretory autophagy, thus limiting bacterial penetration (47). ATG16L1 is recruited to the plasma membrane at the bacterial entry site by NOD2 (48). In addition to autophagy, AMP expression and lysozyme sorting are also regulated by NOD2, suggesting the crucial role of NOD2 in the pathogenesis of CD (49, 50). In Caco-2 cells and the ileum of CD patients, the abnormality of NOD2 is associated with the reduced expression of α-defensins, but not lysozyme (50, 51). *ATG16L1/XBP1* knockout mice develop higher level of intestinal inflammation than mice with *ATG16L1* or *XBP1* deletion, pointing out the compensatory interaction between autophagy and UPR (16). The loss of XBP1 and pEIF2α, two key components of UPR, impairs PC homeostasis as well (52, 53). These findings suggest that autophagy and UPR in PCs may be potential therapeutic targets for CD.

CD is associated with the decreased expression of caspase-8 and the increased occurrence of necroptosis in PCs (54, 55). PC necroptosis leading to PC loss may be the reason for the reduced AMP expression mentioned above in CD. The expression of mixed lineage kinase domain-like protein (*MLKL*), the executor of necroptosis, is positively correlated to disease activity of CD ileitis (56). Abundant expression of receptor-interacting protein 3 (RIP3), another key hub of necroptosis, is observed in PCs from both humans and mice (55). *Caspase-8*-deleted PCs undergo necroptosis in mice without any treatment, and necrostatin-1 (Nec-1, an inhibitor of RIP1-mediated necroptosis) rescues the PC necroptosis induced by TNF-α (55). CD patients with X-linked inhibitor of apoptosis protein (*XIAP*) mutations display fewer PCs than normal CD patients (57). In *XIAP* knockout mice, PC loss is rescued by *RIP3* silencing and Nec-1 administration intraperitoneally, suggesting that PC necroptosis is the reason for PC loss (57). In addition, ATG16L1 also suppresses necroptosis through maintaining mitochondrial functions in TNF-α-treated intestinal organoids (58). The elevated level of IFN-λ is detected in serum and inflamed ileum of CD patients, and it is mainly located at ileal PCs. IFN-λ treatment enhances *MLKL* expression, thus rendering PCs sensitive to necroptosis (56). PC necroptosis not only weakens the function of PCs, but also might trigger the release of inflammatory medium such as ATP and mitochondrial DNA, thus aggravating inflammation. Strategies to control PC necroptosis may be developed to prevent CD ileitis.

CD can induce robust cell apoptosis in crypt regions of ileum (59). Recent study has demonstrated that PCs acting as phagocytes remove the apoptotic cells in intestinal crypts, avoiding the occurrence of inflammation (60). Therefore, PC loss in CD may weaken the engulfment and removal of apoptotic cells in crypts, which requires further experimental demonstration. The accumulation of apoptotic cells in crypts may also lead to inflammation, thus impairing ISC niche and aggravating CD.

In addition to genetic factors, environmental risk factors such as smoking, western diet and, alcohol play an important role in CD pathogenesis (61). *ATG16L1*-mutated CD patients display more abnormal PCs after smoking (62). The treatments of these environmental risk factors in mice lead to PC dysfunction in manners of microbiota alterations, ER stress induction and AMP inhibition (63–65). Activating transcription factor 4 (ATF4) is down-regulated in the inflamed intestine from CD and UC patients (66). ATF4 is responsible for the uptake of glutamine promoting AMP expression, suggesting the importance of alimentary supplementation in IBD (66). However, the involvements of PCs in CD induced by environmental risk factors are poorly understood and still requires further investigation in CD patients. The detailed information about the association between PCs and CD is provided by Wehkamp and Stange (67).

## PCs in necrotizing enterocolitis

NEC is a common gastrointestinal disease with devastating disorders and contributes to high morbidity and mortality in preterm infants, and it is characterized by pneumatosis intestinalis mediated by bacteria-derived gas (68). The pathogenesis of NEC is attributed to intestinal injury and damage that induced by bacterial permeation across the undeveloped epithelial mucosa (18). The decrease or absence of PCs is observed in inflamed intestine from NEC infants, indicating the possibility that PC defects could allow bacterial invasion in NEC (69–71). In addition, PC-derived EGF seems to be beneficial to alleviate NEC since it can reduce intestinal autophagy and NEC incidence in rats (72, 73). Dithizone (a selective destroyer of PCs)-mediated PC loss in combination with acute *Klebsiella pneumoniae* infection induces severe intestinal injury similar to human NEC in immature mice (74). Notably, this method is only applied to postnatal day 14-16 (P14-P16) mice rather than P5 and P28 mice. The limitation may attribute to the fact that PC-dependent innate immunity is essential for the immature intestine of P14-P16 mice (75), highlighting the importance of PCs in preventing NEC. The immature intestine of P5 mice without PCs is protected by cathelin-related antimicrobial peptide (CRAMP), and P28 mice possess mature small intestine (75). In this mouse NEC model, dithizone and *Klebsiella pneumoniae* can be substituted with diphtheria toxin/PC-DTR mice and other bacteria (*Klebsiella Zea mays* and *Bacillus cereus*) respectively (18). PC deletion-induced NEC model mice display the increased *Enterobacteriaceae* species participating in human NEC development, as well as the decreased *Helicobacteraceae* species in cecum (10).

Antibiotic is generally used to treat infection. However, the prolonged antibiotic exposure to preterm infants elevates the NEC incidence (76, 77). Subsequent study conducted with neonatal mice has demonstrated that intraperitoneal antibiotic treatment after birth (P1-P10) reduces PC number in crypts, and *Klebsiella pneumoniae* infection at P14 triggers NEC-like intestinal injury in mice after 10-day antibiotic treatment, suggesting the necessity to control antibiotic use in newborns (78). Efforts to protect intestinal PCs in infants must be conducted to prevent NEC attack.

## PCs in intestinal infection

PCs residing in crypt bottoms are sensitive to intestinal microorganisms mainly including bacteria, virus and parasite. Intestinal bacteria directly stimulate the expression of AMPs *via* toll-like receptor (TLR)-MyD88 axis (79). There are multiple TLR ligands in microorganisms, and their abilities to trigger degranulation of PCs are different. Polyinosinic-polycytidylic acid (TLR3 agonist) and CpG-oligodeoxynucleotide (TLR9 agonist) treatments induce dramatic degranulation of PCs in mice, while LPS (TLR4 agonist) and flagellin (TLR5 agonist)-induced degranulation is dilatory and TNF-α-dependent (80). This degranulation process of PCs also occurs in intestinal organoids (81). Under infectious conditions, lysozyme secretion is achieved by secretory autophagy, an alternative secretion approach, rather than degranulation (47). Functional PCs are required for the maintenance of intestinal health, and PC defects render intestine susceptible to microorganism infections (57, 82, 83).

Intestinal microorganisms have various effects on PCs, highlighting the importance of host-microorganism interactions in PC development. Enterotoxigenic *Escherichia coli* and *Salmonella typhimurium* infections lead to the increases in PC number and AMP expression (84, 85). The expansion of PC attributes to Wnt signaling activation (84) mediated by *Salmonella* protein AvrA (86). *Listeria monocytogenes* inhibits intestinal Notch signaling to facilitate PC differentiation (87). In *Clostridium difficile* infection, intestinal epithelial Stat5 signaling activates Wnt/β-catenin signaling in ISCs, thus promoting PC differentiation and intestinal regeneration (88). In human chronic gastritis induced by *Helicobacter pylori* infection, α-defensins secreted by metaplastic PCs in stomach have a bactericidal effect on *Helicobacter pylori*. These findings suggest that bacterial infections lead to PC activation rather than PC defects.

Compared with bacterial infections, viral infections tend to impair PCs. Early simian immunodeficiency virus (SIV) is localized in close proximity to PCs after infection (4). PCs express pro-inflammatory IL-1β impairing intestinal epithelial barrier in response to SIV infection, which precedes the IFN antiviral response, suggesting that PCs may play an important role in amplifying intestinal inflammation (4). Transmissible gastroenteritis virus-infected piglets exhibit PC mitochondria damage that further impairs Notch signaling and ISC functions (89). As for parasite, helminth infection doubles PC number in mice (90), and Toxoplasma gondii infection results in PC mitochondria damage and PC death depending on mTORC1 signaling (91). In consideration of the antibacterial and ISC-supporting roles of PCs, targeting PCs may be a practical strategy to alleviate viral and parasitic infections.

## PCs in liver disease

Liver disease represents one of the major causes of human being death in the world (92). The incidences of nonalcoholic fatty liver disease (NAFLD) and alcohol-related liver disease (ALD) are increasing these years, which results in the morbidity of liver cirrhosis (LC) and even cancer (93–95). The mouse NAFLD model is established with high-fat diet (HFD) treatment, and NAFLD mice exhibit the down-regulated expression of AMPs (19). The reduction of AMPs leads to the emergence of LPS-positive cells in small intestinal mucosa and liver (19). PC disruption induced by vitamin D deficiency increases the abundance of ileal *Helicobacter hepaticus* (a known hepatic pathogen) and bacterial translocation, which worsens hepatic steatosis and inflammation during HFD treatment (96). Oral administration of DEFA5 reduces the abundance of *Helicobacter hepaticus* in ileum and resolves hepatic steatosis and inflammation (96). However, these results are insufficient to demonstrate the role of PCs in NAFLD since vitamin D has extensive functions *in vivo* and vitamin D deficiency can affect other organs in addition to intestine. Contradictorily, PC deletion caused by dithizone alleviates HFD-induced hepatic lipid accumulation by upregulating the abundance of *Bacteroides* (97). *Bacteroides* promotes the biosynthesis of L-methionine and tetrahydrofolate alleviating hepatic steatosis (98, 99). These inverse results suggest that more studies should be conducted to confirm the role of PCs in NAFLD.

Mouse alcoholic hepatitis (AH) model is established with alcohol gavage, whereas the effect of alcohol on PCs is varied among different parts of gastrointestinal tract. The patients with massive alcohol stimulation display the enhanced expression of HD5 and HD6 in metaplastic PCs as well as the activation of Wnt signaling in antrum (100). Chronic alcohol treatment dramatically boosts the number of PCs in the proximal small intestine (63). However, this effect of alcohol is reversed in ileum since the reduced PC granules and AMP expression are observed in the ileum from alcohol-treated mice (101). In addition, AH patients exhibit the elevated plasmatic REG3α level which is further boosted in AH patients died within 30 days (102). REG3α secreted by PCs can maintain intestinal barrier integrity, and its translocation to blood indicates the changed intestinal permeability. Plasmatic REG3α level is correlated with AH severity, hepatic bacterial translocation and inflammation in AH patients, suggesting that REG3α could be regarded as a potential biomarker of AH (102). The involvements of PCs in AH are further confirmed by the facts that α-defensin deficiency and zinc deprivation aggravates intestinal disorders and hepatic inflammation in alcohol-treated mice.

**Figure 1:**
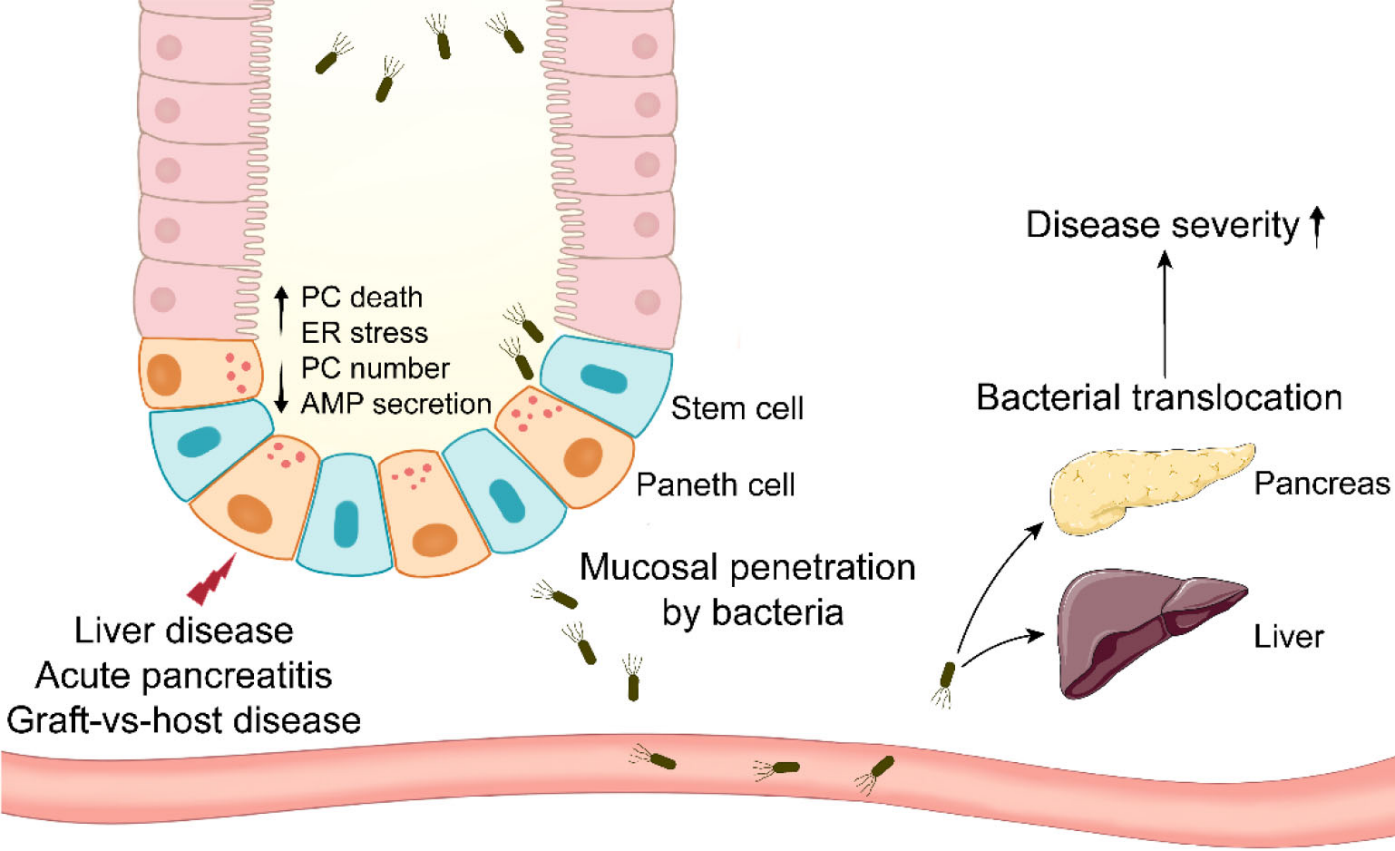
Paneth cells in systemic diseases involving bacterial translocation. Paneth cells are impaired in systemic diseases such as liver disease, acute pancreatitis, and graft-vs-host disease. Paneth cell disruption results in mucosal penetration by intestinal bacteria. The bacterial translocation to systemic organs aggravates the disease severity eventually.

LC is another relatively grievous liver disease. LC patients exhibit the decreased expression of α-defensins and increased plasmatic level of LPS (103). Notably, the α-defensin expression is lower in decompensated LC patients than in compensated LC patients (103). The abnormality of α-defensin leads to hepatic bacterial translocation in LC patients (104). Similarly, hepatic bacterial translocation and the reduced AMP expression are observed in LC rats (105). Recent study has reported that PC disruption is associated with the impaired production of hepatic 25-hydroxyl vitamin D in LC mice, and the loss of intestinal vitamin D receptor aggravates the severity of LC (106). These studies highlight the importance of intestine-liver axis in systematic homeostasis maintenance. HD5 administration and fecal microbiota transplantation mitigate the severe symptoms in LC mice (106). In addition to these major liver diseases, the involvements of PCs in liver are also verified by PC metaplasia in uncommon cystadenomas of liver and extrahepatic bile ducts (107). The decreased AMP expression and enhanced bacterial translocation occur in rats with acute liver failure as well (108). These findings suggest that PCs can secrete AMPs limiting bacterial translocation to alleviate liver diseases (**Figure 1**), highlighting a potential role of PCs in the prevention of liver disease.

## PCs in acute pancreatitis

AP is a potentially lethal disease characterized by its unpredictability and high incidence (109). The meta-analysis has revealed that the global incidence of AP has increased in the late 20th and the early 21st century, particularly in North America and Europe (110). AP patients exhibit the defects in PC number and AMP expression, and similar phenomenon is observed in experimental AP mice and rats (20, 111). In AP rats, intestinal microbiota disorders and PC disruption may be the reasons for the compromised intestinal epithelial barriers, and there is a negative correlation between *Escherichia-Shigella* level and lysozyme expression (111). Hypertriglyceridemia (HTG) is a common risk factor of AP, and it results in approximately 10% incidence of AP attack (112). HTG worsens the intestinal mucosa permeability and AMP expression in AP rats. To examine the role of PCs in AP pathogenesis, dithizone is utilized to disturb PCs in AP mice and rats. Dithizone-induced PC disruption in AP rats triggers severe inflammation through several mechanisms such as ER stress activation, intestinal microbiota alteration and short-chain fatty acids reduction (113, 114). Long-term (2 weeks) deletion of PCs by dithizone exacerbates the inflammation in pancreas and ileum from AP mice, and it leads to an increase in the pathogenic *Helicobacter* and a decrease in the probiotic *Blautia* (20). Although TNF-α from dithizone-treated PCs has been proved beneficial to intestinal cell proliferation, PCs themselves seem to be more important to intestinal homeostasis in AP. Notably, lysozyme administration mitigates the disorders in pancreas, ileal mucosa and intestinal microbiota in AP mice with dithizone treatment. After receiving transplant of feces from lysozyme-treated mice, antibiotic-treated AP mice exhibit the improved symptoms in pancreas and ileum, while transplant of feces from dithizone-treated mice has a reverse effect on the symptoms, suggesting the involvement of PCs in AP *via* intestinal microbiota regulation (20). These studies suggest that stabilizing PCs and microbiota could be a feasible strategy for the therapy of AP (**Figure 1**).

## PCs in graft-vs-host disease

After allogeneic hematopoietic stem cell transplantation, GVHD is a frequent complication due to the immune reaction of allogeneic T cells in transplant against host antigens (115). The allogeneic T cells instinctively attack host intestinal cells, mainly including ISC, goblet cells and PCs (115). The reduced PC number in GVHD patients is correlated with clinical severity and nonrelapse mortality (21). Similar to AH, the level of serum REG3α, a specific GVHD biomarker, is boosted and also correlated with nonrelapse mortality in GVHD patients, whereas the expression of REG3α in small intestine is reduced (116). Therefore, whether there are metaplastic PCs in other sites such as stomach and colon of GVHD patients should be examined in the future work. The patients with severe GVHD (stage 2-4) display lower expression of α-defensins and REG3α in small intestine, while higher expression of them in large intestine, compared to mild GVHD patients (stage 0-1) (117). In GVHD, recipient single nucleotide polymorphisms (SNPs) in DEFA5 (gene for HD5) are involved in GVHD pathogenesis. It is identified that DEFA5 rs4415345G and rs4610776A can effectively prevent GVHD stage 2-4 (118). DEFA5 rs4415345G elevates the abundance of intestinal *Odoribacter splanchnicus* (a butyric acid-producing bacterium), which may decrease the incidence of GVHD stage 2-4 (119). *Odoribacter splanchnicus* can inhibit the production of inflammatory cytokines (120), suggesting the fact that DEFA5 rs4415345G possesses strong anti-inflammatory activity. GVHD mice exhibit the decreases in AMP expression and fecal cryptdin-1 level (121, 122). The reduced α-diversity and abundance of *Escherichia coli* are observed in GVHD mice (121, 122). In addition, the level of *Escherichia coli* is enhanced in MLN and liver from GVHD mice, and antibiotic treatment significantly alleviates the severity of GVHD (122) (**Figure 1**).

In view of the crucial role of PCs in GVHD, treatments targeting PCs may be effective methods to attenuate GVHD severity. This hypothesis is confirmed by direct REG3γ or cryptdin-4 supplementation and IL-22 treatment (in a REG3γ-dependant manner) (116, 123). R-Spondin1, a Wnt signaling agonist, can promote PC differentiation to relieve GVHD (123). A decrease in glucagon-like peptide 2 (GLP-2) derived from intestinal L cells is observed in GVHD patients and mice, and teduglutide (a GLP-2 agonist) treatment facilitates PC regeneration, thus benefiting AMP expression and microbiota control against GVHD (124). In contrast to the enhanced level of IFN-λ inducing PC necroptosis in IBD, IFN-λ in GVHD has no effect on PCs, suggesting the ambiguous role of IFN-λ on PCs under different conditions (125). The novel therapeutic strategies on PCs or AMPs could be effectively applied in GVHD treatment.

## PCs in diabetes

Diabetes serves as a widespread danger to public health, and the morbidity of diabetes is dramatically ascending all over the world. Diabetes is associated with insufficient insulin secretion and insulin resistance (126). Increasing evidence has demonstrated that diabetic patients are susceptible to intestinal pathogen infections (127–129). However, the alterations of PC-derived AMPs in diabetic mice are different in several studies. On one hand, streptozotocin (STZ)-induced diabetes impairs AMP expression in both proximal and distal small intestine, thus leading to the increased bacterial burden and lowered bactericidal activity in intestine (130). The deficiency of endogenous insulin may inhibit AMP expression in diabetic mice since exogenous insulin treatment restores AMP expression (130). On the other hand, STZ-treated mice display enhanced mRNA and protein levels of lysozyme, which attributes to the impaired signal transduction of Notch1/NICD in the small intestine (131). PC number is boosted in small intestine from diabetic mice, and the mechanisms involve the inactivation of Notch/Hes1 signal pathway in ISCs and the activation of insulin receptor-A isoform in PCs (130–132). Further investigation has revealed that the number of Lgr5 positive ISCs is increased in STZ-induced diabetic mice, and Lgr5 positive ISCs isolated from diabetic mice can differentiate into larger proportion of PC lineage compared to those isolated from control mice (133). In addition, insulin resistance induced by S961, an effective antagonist of insulin receptor, impairs AMP expression and granule integrity in PCs, thus leading to the enhanced intestinal permeability and the occurrence of low-degree inflammation (134). Although diabetes involves PCs, the role of PCs in diabetes is still indistinct and requires massive investigation in clinical trials and mice with PC deletion or AMP treatment.

## PCs in obesity

Obesity has become a worldwide epidemic during the last few decades. The current prevalence of obesity in America is 18.5% among youth and 39.6% among adults (135), and that in Europe is 15.3% to 25.6% among youth (136). Obese people tend to exhibit PC abnormality (64, 137). HFD-induced obesity triggers PC defects, microbiota composition alterations and low-grade intestinal inflammation in mice, and the alterations of PCs and microbiota occur prior to intestinal inflammation (138).

PCs possess abundant ER and high protein biosynthesis activity to support their highly secretory nature, and PCs are susceptible to ER stress triggered by the accumulation of misfolded or unfolded proteins in ER (139). ER stress can induce UPR activation facilitating the restoration of ER homeostasis. Obese people display the reduced protein levels of HD5 and lysozyme and the elevated gene expression of them (137). Notably, the activated UPR and ER stress are present in the jejunal PCs from obese people, and UPR activation is negatively correlated with lysozyme level, suggesting that the impaired protein biosynthesis of AMPs is the reason for the discordance between AMP protein level and mRNA expression (137).

Bile acid plays an important role in HFD-induced PC defects. HFD treatment enhances the level of bile acid, and bile acid can bind to G protein-coupled bile acid receptor (TGR5) highly expressed on the membrane of PCs (140). The elevated bile acid induce ER stress to impair PC functions and AMP expression, which can be rescued by pretreatment with ER stress inhibitor 4PBA or bile acid binder cholestyramine (140). Certain bacteria, such as *Clostridium* spp., are major sources of deoxycholic acid (DCA) and lithocholic acid (LCA) that are enriched in the ileum of western diet or HFD-treated mice (64, 140). *Clostridium*-mediated DCA production activates farnesoid X receptor (FXR) pathways in PCs and myeloid cells, which leads to PC defects (64).

**Figure 2:**
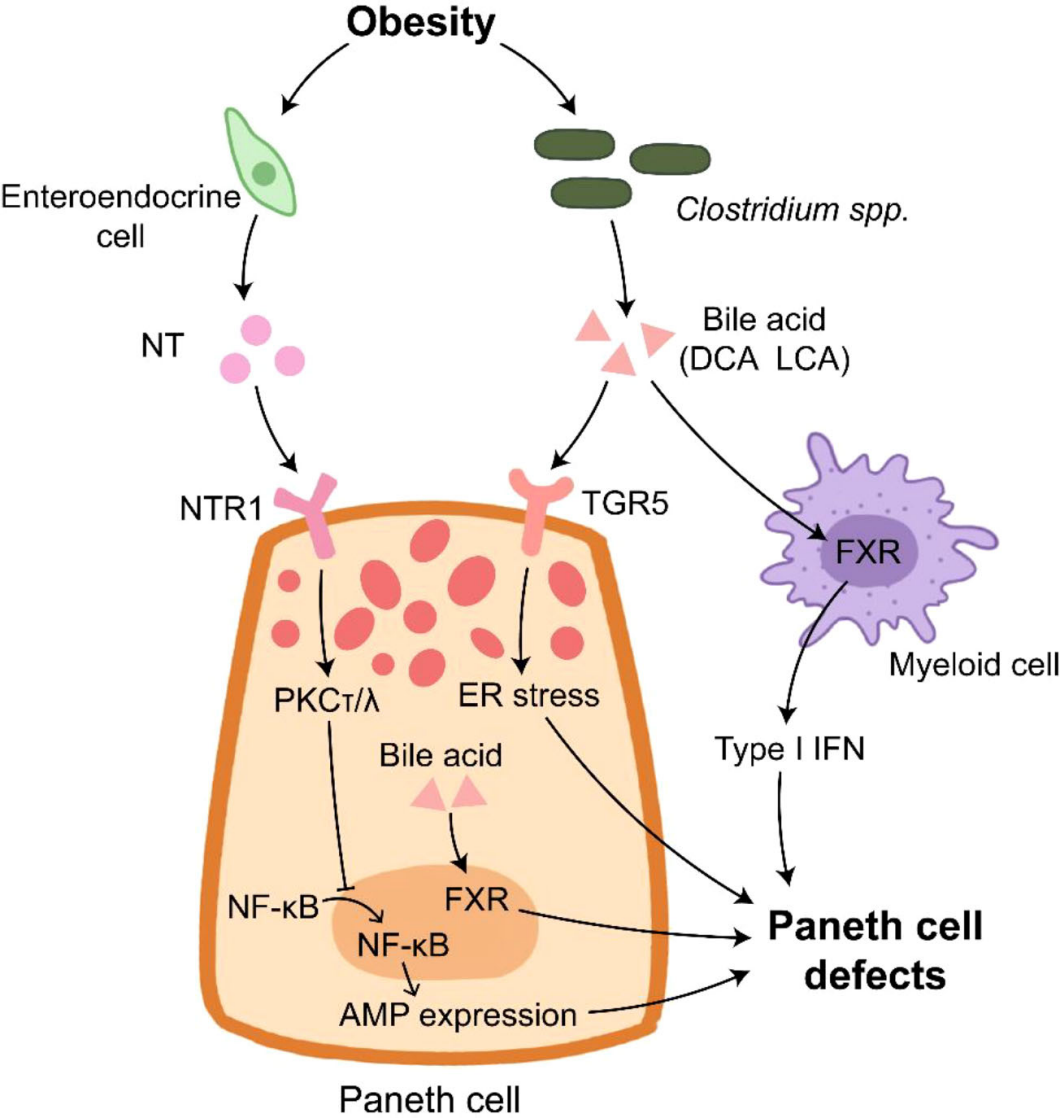
Paneth cells in obesity. Paneth cell disruption in obese individual is under multiple control. NT derived from enteroendocrine cells inhibits AMP expression through inducing PKCτ/λ that suppresses the translocation of NF-κB into nucleus. The elevated bile acid provided by *Clostridium* spp. binds to TGR5 to induce ER stress in Paneth cells. In addition, bile acid activates FXR in Paneth cells and myeloid cells, which results in Paneth cell defects.

Plasma neurotensin (NT), an enteroendocrine cell-derived hormone, is also involved in HFD-induced PC defects since NT deficiency alleviates the impaired PC function in HFD-treated mice (141). NT binding to NT receptor 1 (NTR1) activates PKCτ/λ to inhibit the nuclear translocation of p65, thus impairing AMP expression. Besides, the deletion of intestinal epithelial insulin receptor decreases the elevated AMP mRNA expression induced by HFD treatment, but it has no effect on the number of lysozyme positive cells in jejunum (142). These findings suggest the impaired function of PC under obese condition (**Figure 2**). HD5 treatment reduces circulating cholesterol and fatty acids in obese mice (143), implying the feasibility that utilizes AMP as a complemental method for obesity therapy.

## PCs in ischemia reperfusion

**Figure 3:**
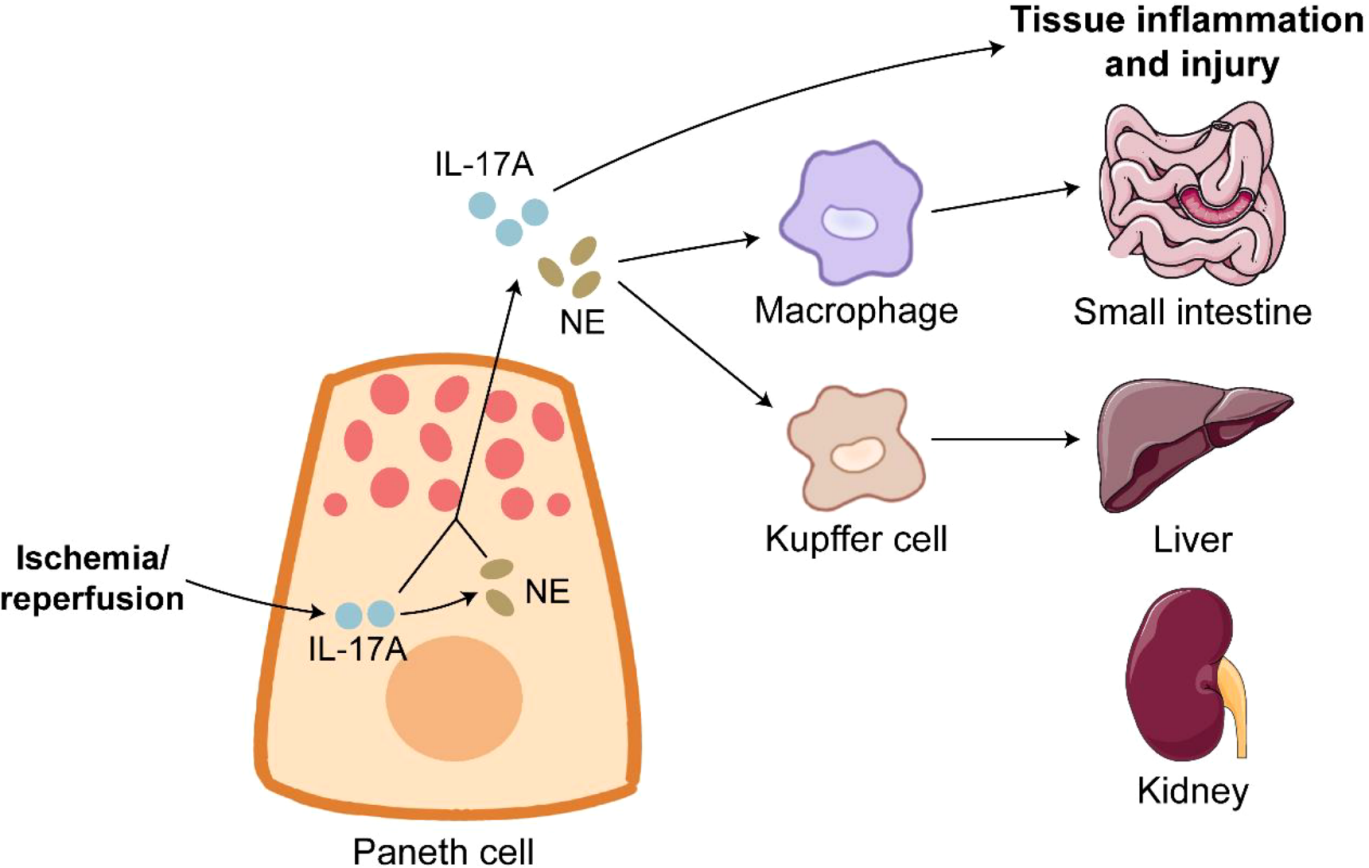
Paneth cells in ischemia/reperfusion. Ischemia/reperfusion triggers robust degranulation in Paneth cells. IL-17A release from Paneth cells directly leads to inflammation and injury of organs such as small intestine, liver and kidney. Furthermore, IL-17A in Paneth cells promotes the expression of NE. NE binds to its receptor on macrophages and Kupffer cells to induce tissue inflammation and injury.

IR is a serious condition of prolonged inadequate organic blood supply and subsequent sudden restoration of blood flow, and IR causes catastrophic and deadly injury to many organs such as intestine, liver and kidney (144, 145). Approximately 30% deaths of ischemic patients attribute to IR injury (146). UPR activation and PC apoptosis induced by ER stress are observed during intestinal IR injury in humans and rats, and PC disruption by dithizone further exacerbates intestinal epithelial permeability and inflammation in the intestine of IR rats (147). IR injury dramatically induces PC degranulation, and IL-17A in PC granules is responsible for multi-organ injury during IR. IL-17A neutralization or PC deletion protects organs from inflammation and injury induced by IR (6, 148). The detrimental role of PC-derived IL-17A in IR is achieved by macrophage-mediated transportation (149). PC hyperplasia induced by *TLR9* deletion worsens multi-organ inflammation and injury after IR in an IL-17A-dependant manner (150, 151). As expected, intravenous treatment of IL-17A neutralizing antibody effectively alleviates IR-induced severe inflammation and injury in *TLR9* knockout mice (150). The expression of tyrosine hydroxylase, a key enzyme of norepinephrine (NE) synthesis, is detected in human and mouse PC. NE release is driven by IL-17A in PCs (152). NE activates intestinal macrophages and Kupffer cells to damage multiple organs after IR, and the block of α-adrenergic receptor significantly alleviates IR-induced injury in mice (152). The modulation of PC-derived IL-17A and NE could have therapeutic value for the treatment of IR-mediated systemic complications (**Figure 3**).

## PCs in COVID-19

**Figure 4:**
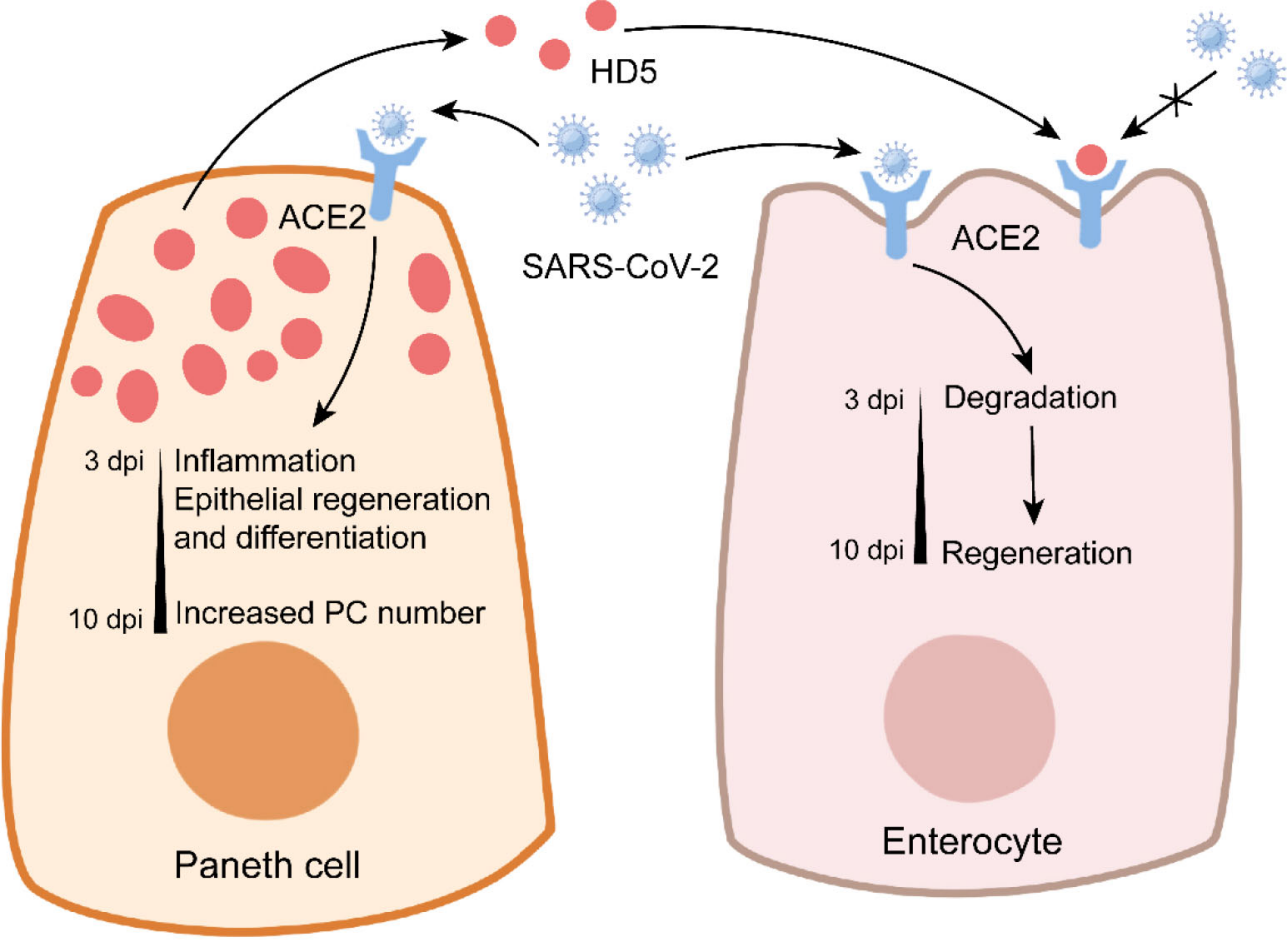
Paneth cells in COVID-19. In Paneth cells, SARS-CoV-2 infection triggers the expression of genes related to inflammation and epithelial repair, and it enhances the number of Paneth cells at later stage. In enterocytes, SARS-CoV-2 infection leads to the conversion from degradation to regeneration over time. Notably, Paneth cell-derived HD5 binds to ACE2 to prevent SARS-CoV-2 infection.

COVID-19 caused by severe acute respiratory syndrome coronavirus 2 (SARS-CoV-2) has rapidly risen to a threatening and lethal epidemic worldwide (153). Although COVID-19 is a respiratory disease characterized by cough and severe pneumonia, gastrointestinal dysbiosis such as diarrhea and abdominal pain also occurs in COVID-19 patients (154, 155). SARS-CoV-2 infection inhibits ZO-3 and claudin-1 expression to impair intestinal epithelial integrity (156). PCs express certain genes related to SARS-CoV-2 entry, such as angiotensin-converting enzyme 2 (ACE2) and serine protease transmembrane protease 2 (TMPRSS2), thus rendering PCs susceptible to SARS-CoV-2 infection (157). SARS-CoV-2 mainly targets enterocytes and PCs in the intestine (156). SARS-CoV-2 infection increases PC number in the small intestine of rhesus macaques at 7-10 dpi (158). PCs exhibit the activated gene expression of factors related to cell cytoskeleton organization and epithelial cell differentiation at 3-7 dpi, which may contribute to the enhanced expressions of ZO-1 and claudin-1 at 10 dpi, suggesting the important role of PCs in intestinal epithelial repair during SARS-CoV-2 infection (158) (**Figure 4**). However, PC-mediated epithelial repair leads to rectal viral shedding accelerating viral transmission (158). In addition, aging PCs disturb intestinal ISC functions, thus indirectly impairing the differentiation of M cells (159). This may explain why the olds are more susceptible to COVID-19 than the youths (160).

HD5 plays a critical role in the inhibition of SARS-CoV-2 infection (**Figure 4**). As the most abundant α-defensin secreted by PCs, HD5 can bind to the ligand-binding domain of ACE2 with a high affinity (161). In Caco-2 cells, HD5 pretreatment decreases SARS-CoV-2 binding to ACE2, suggesting the competition for ACE2 between HD5 and SARS-CoV-2 (161). It is worth noting that HD5 has no protective effect on SARS-CoV-2 infection when provided post-infection or as precursor form (162). These studies suggest the beneficial role of PCs in COVID-19. More information about the effect of AMPs (from PCs or not) on SARS-CoV-2 is recently discussed by Ali et al. in detail (163).

## PCs in portal hypertension

Portal hypertension (PH) is associated with the increased portal pressure induced by elevated resistance of blood stream (164). Blocking intestinal angiogenesis has a beneficial effect on the alleviation of PH (165). The reduced intestinal angiogenesis and PC number in GF mice imply a potential association between PCs and PH (166). PC deletion by dithizone dramatically decreases the angiogenesis in small intestinal homeostasis (167). Mouse PH model is established with partial portal vein ligation (PPVL). Hassan et al. firstly reported that portal pressure and portosystemic shunts are weakened in PC-deleted mice after PPVL (168). PC disruption weakens intestinal and mesenteric angiogenesis in PPVL mice, which attributes to the reduced expression of angiogenic genes (168). Furthermore, intestinal microbial signals are responsible for the induction of angiogenic factors derived from PCs, thus promoting the angiogenesis of endothelial cells (168). In addition to angiogenesis, lymphangiogenesis is also supported by PCs (169). During PPVL, PCs secrete lymphangiogenic factors to facilitate intestinal and mesenteric lymphangiogenesis in response to intestinal microbial signals (169). These findings suggest that PC could be a potential target for therapeutic interventions of PH.

## Therapeutic strategies targeting PCs in various diseases

**Figure 5:**
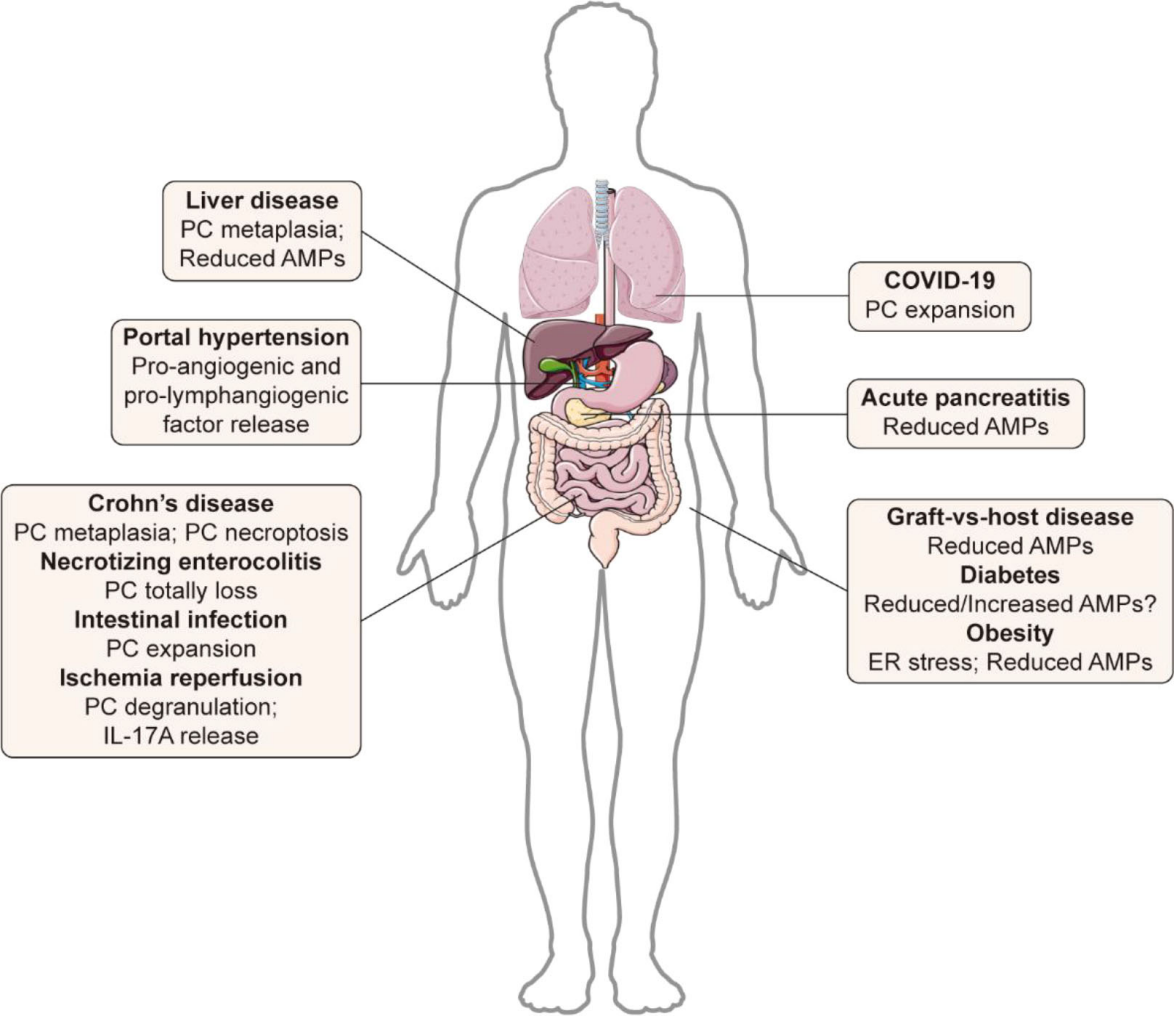
Differences in PC performance in various diseases. Paneth cells exhibit different responses to diseases. The most characteristic features of Paneth cells under different pathological conditions are summarized in the figure.

As mentioned above, PC defects are involved in many diseases within intestine and systemically, which worsens the severity of diseases. However, PC performance can be different in these diseases (**Figure 5**), suggesting that the therapeutic strategies should be also flexible. Therapeutic strategies targeting PCs mainly include three aspects: PC protection, PC-derived inflammatory cytokine elimination, and substituting AMP treatment.

PC protection is aimed to restore PC homeostasis affected by many factors such as mitochondrial abnormalities, ER stress, and cell death. Mitochondrial abnormalities are observed in PCs of CD patients (33). Mito-Tempo (a mitochondrial-targeted antioxidant) treatment improves the inflammatory response, metabolism, apoptotic, and epithelial barrier function in the ileal biopsies from CD patients (33). ER stress in PCs is generally induced in many diseases, which leads to the abnormality of PCs (170). Efforts to inhibit ER stress could be conducted to alleviate disease severity. In obese individuals, bile acid is also an alternative target due to its ability to trigger ER stress in PCs (140). Excessive ER stress can induce PC apoptosis (171), thus lessening the number of PCs. Besides, several risk factors in CD, such as IFN-λ and *ATG16L1*, are able to induce PC necroptosis (56, 58). Uncontrolled cell death of PCs not only leads to the loss of PCs directly, but also induces the occurrence of intestinal inflammation. Therefore, methods to preserve the functional PCs could be an effective therapy, such as the administration of drug inhibiting necroptosis and alleviating ER stress.

Although PCs play an important role in intestinal homeostasis, the functional PCs seem to be detrimental to health in some cases. IL-17A and IL-1β are two pro-inflammatory cytokines in the granules of PCs. IR triggers the degranulation of PCs and the subsequent release of IL-17A responsible for multi-organ injury (6, 148). IL-17A neutralization or PC deletion effectively attenuates IR-induced injury (148), suggesting the feasibility of blocking the production of IL-17A in IR. In SIV infection, IL-1β production in PCs is rapidly conducted, and it is prior to AMP expression in PCs and type 1 IFN response in intestinal mucosa, suggesting that PC-derived IL-1β may be the key origin of inflammation (4). The presences of activated IL-1β and caspase-1 (a key component of pyroptosis) is also observed in PCs after irradiation (172). However, whether IL-1β neutralization or PC pyroptosis inhibition could weaken the amplification of intestinal inflammation in these diseases still remains unclear.

Since PCs can regulate the composition of intestinal microbiota *via* AMPs, these diseases impairing PCs generally accompany with the disorders in microbiota. For example, the abundances of pathogenic bacteria *Ruminococcus gnavus* and adherent-invasive *Escherichia coli* are elevated in ileal lumen from CD patients (41, 173), and the increased pathogenic bacteria *Helicobacter* and the reduced probiotic bacteria *Blautia* are observed in the ileocecum of AP mice (20). To improve the disordered microbiota and disease severity, fecal microbiota transplantation has been proved feasible according to the results from research on AP mice (20). PC disruption in combination of microbiota disorders results in bacterial translocation aggravating the severity of NEC, AH and AP (68, 101, 112). In most diseases involving PC disruption, PC-derived AMP treatment significantly alleviates the symptoms of diseases, suggesting the importance of PC-derived AMPs in controlling intestinal microbiota. Furthermore, utilization of antibiotic to destroy the intestinal microbiota is another effective strategy on GVHD treatment (122). However, antibiotic is not applicable to NEC since antibiotic-induced PC disruption renders intestine susceptible to NEC attack in newborns (78).

## Conclusion

PCs are known as the guardians of the small intestine. In this paper, we introduce the multifaceted involvements of PCs in intestinal and extraintestinal diseases. Large amounts of systemic diseases such as CD, NEC and COVID-19, are associated with PC disruption. PCs are involved in these diseases *via* various mechanisms. PCs possess numerous risk genes related to CD, such as *ATG16L1* and *XBP1*. In addition, PC-mediated limitation of intestinal bacterial translocation is of importance for the prevention or alleviation of diseases. However, the role of PCs in IR is different from other diseases. The presence of PC-derived IL-17A aggravates the multi-organ injury induced by IR, and the removal of IL-17A or PCs has a protective effect in IR. In COVID-19, PCs can inhibit the entry of virus and promote intestinal regeneration to resist SARS-CoV-2 infection. Except for IR, AMP treatment has a beneficial effect on all diseases involving PCs. All in all, strategies to stabilize PCs could be developed to effectively intervene these diseases within intestine and systemically.

## Author contributions

Writing - original draft: CC. Visualization: XW, LL, and HW. Writing - review & editing: CC and JP. All authors contributed to the article and approved the submitted version.
